# Short time to positivity of blood culture predicts mortality and septic shock in bacteremic patients: a systematic review and meta-analysis

**DOI:** 10.1186/s12879-022-07098-8

**Published:** 2022-02-10

**Authors:** Ya-Chu Hsieh, Hsiao-Ling Chen, Shang-Yi Lin, Tun-Chieh Chen, Po-Liang Lu

**Affiliations:** 1 Kaohsiung Medical University Hospital, Kaohsiung Medical University, Kaohsiung, Taiwan; 2 Department of Pharmacy, Kaohsiung Municipal Ta-Tung Hospital, Kaohsiung Medical University, Kaohsiung, Taiwan; 3 Division of Infectious Diseases, Department of Internal Medicine, Kaohsiung Medical University Hospital, Kaohsiung Medical University, Kaohsiung, Taiwan; 4 Department of Laboratory Medicine, Kaohsiung Medical University Hospital, Kaohsiung Medical University, Kaohsiung, Taiwan; 5 School of Medicine, College of Medicine, Kaohsiung Medical University, Kaohsiung, Taiwan; 6 Department of Internal Medicine and Infection Control Office, Kaohsiung Municipal Ta-Tung Hospital, Kaohsiung Medical University, No. 68, Chunghwa 3rd Road, Kaohsiung, Taiwan

**Keywords:** Time to positivity, Patient outcome, Mortality, Septic shock

## Abstract

**Background:**

The value of time to positivity (TTP) on diagnosis for catheter-related bloodstream infection and distinguishment on bacteria group and infection source has been investigated. However, the relationship between TTP and patient outcome requires verification, and we performed a systematic review and meta-analysis.

**Methods:**

We searched PubMed, EMBASE, CINAHL, Cochrane Library, Web of Science for publications associated with the topic. We included studies that researched the TTP on predicting patient mortality and septic shock. Quality assessment is performed with Critical Appraisal Skills Programme (CASP). The analysis is performed using Review Manager Version 5.0.24. on articles available for data extraction on the exact population of each outcome group. The existence of publication bias was assessed by funnel plots. Statistical heterogeneity was evaluated using the Cochran Q and ${I}^{2}$ statistics. The outcome is reported as an odds ratio. PROSPERO registration: CRD42021272286.

**Results:**

Twenty-four eligible studies were included in our study. Twenty-four in the mortality group and six in the septic shock group. Mortality is significantly associated with the short time to positivity group with an odds ratio of 2.98 (95% CI: 2.25–3.96, p-value < 0.001). The odds ratio for developing septic shock in the short TTP group is 4.06 (95% CI: 2.41–6.84, p-value < 0.001). Subgroup analysis revealed short TTP as a significant predictor of mortality and septic shock in *Gram's* positive and *Gram's* negative related bloodstream infections. TTP is not associated with mortality among patients with candidaemia.

**Conclusions:**

Short time to positivity is a reliable marker for patient outcome in certain bacterial species. Studies concerning confounding factors such as the delay in bottle loading and other confounding factors are needed to enhance external validity.

**Supplementary Information:**

The online version contains supplementary material available at 10.1186/s12879-022-07098-8.

## Background

Time to bacterial culture positivity, or time to positivity (TTP), is defined as the time from the start of incubation to the preliminary positive result of blood culture. The value of TTP provides indirect information of bacteremia load in the blood sample and is perceived as a new method for physicians to identify or evaluate the treatment or prognosis of the patient [1, 2]. Initially, the utility of TTP is focused on recognizing the bacteria genre or infection source. TTP was then reported to be helpful with differential diagnosis of catheter-related bloodstream infections (CRBSIs) [3], salvaging central venous catheters [4], and prognosis prediction for infective endocarditis [5]. Short TTP had also been acknowledged to be associated with mortality [5–9].

Though an increasing number of TTP-associated articles were published in the past decade, there is still a notable knowledge gap on the outcome prediction of TTP. Despite many articles had analyzed the correlation of TTP and patient outcome, the application of TTP requires further study. Most of the studies faced limitations such as small study populations, uncontrolled confounders, and differentiation between centers [10]. These hurdles make the implementation of TTP impractical. Thus, we aim to clarify the relationship between TTP and patient outcome, test the robustness of TTP as a predictor in patient outcome, and overcome the limitation of population size in current articles by performing a systemic review and meta-analysis.

## Methods

### Search strategy and study selection

We followed the Preferred Reporting Items for Systematic Reviews and Meta-Analyses (PRISMA) statement to conduct this systematic review. [11] We searched PubMed, EMBASE, CINAHL, Cochrane Library, Web of Science through August 13, 2021. The search was done with Medical Subject Headings (MeSH), and appropriate adjustments were made according to different databases. The index terms included time to positivity, time to blood culture positivity, mortality, septic shock, and prognosis, Supplementary Material Table gives the retrieval strategy in detail. We included prospective and retrospective observational studies that addressed: 1) Association of the length of TTP and patient prognosis (mortality and septic shock) or 2) Cut-off value of TTP for patient prognosis prediction. The search incorporated a limitation for articles available in English after 2000 and studies involving humans. Case reports and reviews were excluded. This study is registered with the International Prospective Register of Systematic Reviews (PROSPERO) as record number CRD42021272286.

Two authors assessed the titles and abstracts of the studies to eliminate those not relevant to the inclusion criteria. Eligible studies include those focused on patient's blood culture TTP's impact on patient outcome (mortality and septic shock). Articles discussing TTP after antibiotic usage were excluded.

### Data extraction

Two independent reviewers (YC Hsieh and TC Chen) extracted the data using a standardized protocol and PICO (Patient, Intervention, Comparison, and Outcome). Disagreement on specific studies between the two reviewers was resolved through discussion or consultation with the third reviewer (SY Lin). Quality assessment is performed with the Critical Appraisal Skills Programme (CASP). The titles and abstracts were screened for relevance. After a review of the full-text articles, the following data were extracted from each study: the year of the publication, patient characteristic, study design, TTP characteristic, the proportion of death and survival in both short and long TTP groups, and the proportion of patients developing septic shock in short and long TTP groups. Short TTP and long TTP are defined through the cut-off value in each article. TTP shorter than the cut-off value defined in each study is classified into short TTP group, vice versa. The classification of septic shock was defined according to the criteria previously published in The Journal of the American Medical Association (JAMA). [12] Only articles using this criterion for septic shock were included in our analysis.

### Statistical analysis

The exact number of mortality events and septic shock events in both short TTP and long TTP groups extracted from each study were combined using a Mantel–Haenszel statistical method with random-effects model, which assumes that individual studies are estimating different treatment effects, rather than the fixed-effect model, which is based on the mathematical assumption that a single common effect underlies every study in the meta-analysis. We performed the statistical analysis and subgroup analyses based on *Gram’s* stain and *Candida spp.* on mortality and septic shock, respectively. In order to eradicate the bias effect contributed from the variation of cut-off value defined in each article included in the mortality group, we conducted another statistical analysis based on studies with defined TTP cut-off values shorter than the median in the mortality group, which is 12 h. Dichotomous data were reported as an odds ratio with corresponding 95% CI p-value < 0.05 is considered to be significant. Statistical heterogeneity was evaluated using the Cochran Q and ${I}^{2}$ statistics. '${I}^{2}$' denotes the percentage of total variation across the studies that are the result of heterogeneity rather than chance. We assessed for the presence of publication bias using a funnel plot. The meta-analysis was performed using Review Manager Version 5.0.24.

## Results

**Fig. 1:**
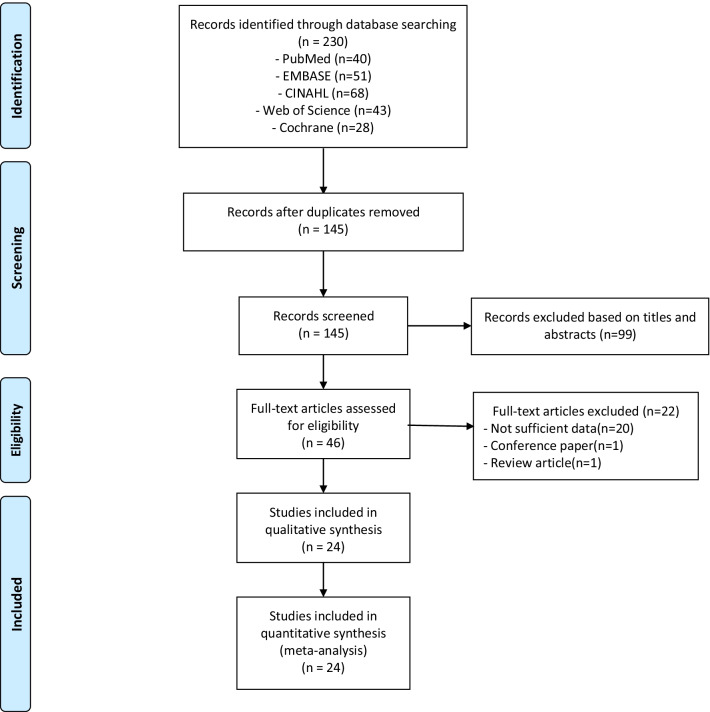
Flow diagram of the study selection

The initial database search identified 230 articles. We screened titles and abstracts of a total of 145 non-duplicate records and excluded 99 articles not relevant to the topic. A total of forty-six full-text articles were reviewed for eligibility, and twenty-four studies that met our search criteria with eligible quality were included in the analysis. Twenty-four studies with complete data (twenty-four for patient mortality and six for patients developing septic shock) were included in the final meta-analysis (Table 1). All studies included in our analysis are based on bacteremia. Detailed results of our search are presented in Fig. 1 as a PRISMA flowchart.

**Table 1:** Table of included studies

Year, author	Country	Bacteria	Culturing method	Study design	Cut-off value (hrs)	Population	Patient Characteristic
2006, Alexandre R. Marra et el. [13]	USA	*Staphylococcus aureus*	Blood cultures were processed by the institution's clinical laboratory using the BacT/ALERT blood culture instrument. All the bottles were loaded into the instrument at any time of the day (24 h a day, 7 days a week) without delay	Historical cohort study	12	91	A total of 91 patients with monobacterial S. aureus BSIs with no antibiotics treatment were identified at VCUMC were retrospectively by use of the electronic medical microbiology record
2006, Jose A. Martı´nez et al. [16]	Spain	*Escherichia coli*	Blood cultures were processed by the BACTEC 9240 system. The volume of blood cultured was not verified. The exact time from the start of incubation to a positive reading was recorded, considering time to positivity to be that for the bottle in the processed set or sets that became positive first	Prospective study	7	177	During one year (2003), all 177 patients with 185 episodes of E.coli bloodstream infections were prospectively followed up from diagnosis to discharge
2007, G. Peralta et al. [17]	Spain	*Escherichia coli*	They took 20 mL of venous blood and inoculate it in equal parts into one aerobic blood culture bottle and one anaerobic blood culture bottle. Blood from a peripheral vein was sampled by nurses, three times at intervals of 30 min. On a 24-h basis, the blood culture bottles were sent to the microbiology laboratory, immediately loaded into the blood culture instrument (BacT/ALERT microbial detection system; bioMe´rieux), and cultured for 5 days. The BacT/ALERT system tests for CO2 production and records the time interval between the addition of each blood culture bottle to the system and the detection of microbial growth (defined as TTP). No antibiotic removal device was used for the blood cultures of patients treated previously with antibiotics. When multiple cultures were positive, the shortest TTP was selected for the analysis. Neither the volume of blood cultured nor the time interval between obtaining the blood for culture and incubation of the bottles was recorded	Retrospective study	10.25	459	459 cases of monomicrobial *E. coli* bloodstream infections with no undergoing antibiotics treatment from a single institution between 1997 and 2005 were reviewed
					11.1		
2009, C.-H. Liao et al. [32]	Taiwan	*K. pneumoniae*	At least two sets of blood samples, 10 mL each, were taken from separate locations, and inoculated into aerobic and anaerobic culture flasks, which were incubated using the BACTEC 9240 automated detection blood culture system. The BACTEC 9240 system continuously monitors CO2 production every 10 min, and indicates positivity by means of a fluorescent signal. All bottles with positive results were examined by Gram staining, and subcultured. TTP, defined as the time from the start of incubation to the start of the alert signal, was recorded for each bottle of positive blood cultures. For a patient with multiple sets of positive blood cultures at approximately the same time, the shortest TTP was used in the study	Prospective cohort study	7	231	Patients > 18y/o admitted with monomicrobial K. pneumoniae BSI
2010, J. Kim et al. [8]	Canada	S*taphylococcus aureus*	All blood cultures were obtained by use of a standardized sterile technique. Upon receipt, the blood culture bottles were placed in BacT/ALERT 3D blood culture system at any time of the day. Each blood culture set consisted of an aerobic and anaerobic bottle with a volume of 10 mL per bottle. Time to positivity was defined as the first bottle in a set to be flagged positive. If multiple sets were inoculated in the same 24-h period, the earliest time at which a bottle was flagged positive was used as the TTP	Retrospective study	48	684	All persons identified in the Calgary Health Region with first episode S. aureus bacteremia between July 1, 2006 and December 31, 2008 were included in the study
2011, Mahesh B Savithri et el. [29]	Australia	Coagulase-negative staphylococci	Blood samples were collected using aseptic technique. 20 millilitres of blood were collected and inoculated in two equal aliquots of 10 mL each into a BacT/ALERT aerobic bottle and an anaerobic bottle and incubated at 37 °C. Organisms isolated from blood culture bottles were identified by the automated BacT/ALERT system	Retrospective study	24	54	A retrospective chart audit in the intensive care unit of a tertiary hospital comprising all patients who had positive blood cultures for CoNS in 2009
2012, R. Álvarez et al. [18]	Spain	*Escherichia coli*	Following the hospital protocol, the blood culture bottles were sent to the microbiology laboratory and immediately loaded into the blood culture instrument (BACTEC FX system; Becton Dickinson), during the day (8 am to 9 pm) or stored during the night until 8 am. They were incubated for a maximum of five days and the TTP was defined as the exact time from the start of incubation to a positive reading	Retrospective study	8	226	All patients > 14y/o with E. coli bacteraemia with at least one positive blood culture were identified retrospectively from the clinical microbiology laboratory database. Only the first positive blood culture was considered, or those which were separated by at least 30 days from the previous one
2013, Cintia Zoya Nunes [24]	Brazil	*Candida albican*	Blood cultures were processed by the institution's clinical laboratory using the BACTEC 9240 blood culture instrument. Each blood culture set consisted of an FA aerobic bottle and an SN anaerobic bottle. All the samples of blood cultures were collected and submitted in a timely manner to the microbiology laboratory. All cultures were obtained via peripheral venipuncture and at least two bottles were obtained for each patient. The bottles were loaded into the instrument (24 h a day, 7 days a week) without delay at any time of the day. The time to positivity of the first bottle in a set to be flagged as positive was used to determine the time to positivity and was obtained by using the system's software	Historical cohort study	36	89	Patients with BSIs from 1 January 2002 through 31 July 2009 were identified retrospectively. Each patient was included only once, at the time of the first BSI. Patients less than 18 years old, those with polymicrobial infections, and those receiving antifungal therapy at the time of the BSI were excluded from the analysis
2013, H. R. Palmer et el. [28]	USA	GNB bacteremia	Blood cultures were processed using the VersaTREK automated blood culture system. This system requires 10 mL of blood to be split between aerobic and anaerobic 40-mL VersaTREK bottles. Inoculated bottles were transported to the microbiology laboratory and placed in the VersaTREK instrument. Bottles were incubated either until they were flagged as positive or for 5 days	Prospective observational study	11	63	Patients > 18 y/o with ≧1 blood cultures positive for GNB
2013, Matthias Willmann et al. [9]	Germany	*Pseudomonas aeruginosa* bloodstream infection	From all patients an approximate volume of 10 ml blood was inoculated into each aerobic and anaerobic BACTEC PLUS bottle and was promptly transported to the microbiological laboratory. Bottles with antibiotics were not used. We generally recommend the concomitant drawing of blood from at least three different peripheral punctures after disinfection. However, in practice we usually received two blood culture sets. Blood cultures were processed employing the Bactec 9240 blood culture instrument. The system monitors bacterial growth by measuring CO2 production through fluorescent sensor technology every 10 min. An automated alert signal indicates a positive blood culture. Time to positivity was documented by the system software and recorded for each bottle from an index culture. The final analysis was performed on the TTP from aerobic bottles since P. aeruginosa grew only in a limited number of anaerobic bottles. Blood culture bottles were usually processed between 8 am and 6 pm during the week and between 8 am and 2 pm during the weekends. Our laboratory provides an on-call service that processes samples during late hours. Thus, transport and storage of samples amounted to less than 12 h in most cases	Retrospective cohort study	15	74	From 2006 until 2012, 74 patients with monomicrobial Pseudomonas aeruginosa blood stream infection were studied in 3 hospitals in the region surrounding Tubingen, Germany
					17		
					18		
2013, Si-Hyun Kim et al. [25]	Korea	*Candida albican*	Blood cultures were performed in patients with signs or symptoms of infection as routine practice. In patients with a positive culture for yeast, blood cultures were usually repeated every 3 days until negative or when indicated clinically. At least two sets of blood samples were obtained for blood culture from separate venepunctures. If a central venous catheter was present, a blood culture set could be replaced by a blood sample drawn through the catheter. Each set of blood samples was inoculated into one aerobic and one anaerobic bottle and immediately loaded into a BacT/ALERTw 3D Microbial Detection System	Retrospective study	24	152	All consecutive patients ≧18 years of age with candidaemia between January 2006 and July 2012 were included
2014, Hui-Wen Lin et al. [22]	Taiwan	*Nontyphoidal samonella*	Two sets of blood samples (10 mL each) were generally taken from separate locations, inoculated into aerobic and anaerobic culture flasks, and then incubated using the BACTEC 9240 automated detection blood culture system. All bottles were loaded when they were received in the central laboratory. The BACTEC 9240 system continuously monitors carbon dioxide (CO2) production every 10 min, and indicates positivity by a fluorescent signal. The TTP, defined as the time from the start of incubation to the start of an alert signal, was recorded for each blood culture. When multiple cultures were positive, the shortest TTP was used for analysis	Retrospective study	10	66	From January 2010 to December 2012, 66 patients > 20y/o with NTS bacteremia were identified by central laboratory personnel. Included only at the time of the first bacteremia
2014, M-S. Hsu et el. [14]	Taiwan	*Staphylococcus aureus*	Blood samples (approximately 10 mL) were taken and inoculated into aerobic and anaerobic culture flasks, and incubated in the automated detection blood culture system. All samples were loaded into the machine upon arrival at the central laboratory. The BACTEC 9240 system monitors CO2 production continuously at 10-min intervals, and positivity is indicated by means of a fluorescent signal. The TTP is automatically recorded by the machine. TTP was recorded for each positive sample. For a patient with multiple sets of positive blood cultures, the shortest TTP was selected. Blood cultures collected from central venous catheters were not excluded	Retrospective study	12	87	From January 2007 to December 2011, we enrolled 87 consecutive patients who had S. aureus bacteraemia persisting for > 48 h at our institution, excluded patients who were aged < 18 years and had polymicrobial bacteraemia were excluded
2016, Qing Zhang et el. [27]	China	Enterobacteriaceae	Blood cultures containing 8–10 mL blood from patients were incubated using an automated blood culture system at 35 °C for at least 5 days. All isolates were identified using the Vitek 2 Compact automated microbiology system	Retrospective study	8	173	From September 2013 through March 2015, all hospitalized tumor patients (> 18 y/o) with at least one episode of BSI were included in the study
		Nonfermenters			18	25	
		Staphylococci			18	49	
2017, Catia Cillo' niz et al. [7]	Spain & Argentina	Pneumococcal	Blood cultures were processed by the BACTEC 9240 system, and vials were loaded into the machine around the clock. Volumes between 8 to 10 ml of blood samples were inoculated into aerobic and anaerobic vials. The incubation period was 5 days before being discarded as negative	Prospective observational study	9.2	278	Including 278 immuno-functioning adults consecutively admitted between 2003 to 2015 with a diagnosis of community-acquired pneumococcal pneumonia to the Hospital Clinic of Barcelona, Spain
2017, Poh-Chang Tang et al. [20]	Taiwan	*Pseudomonas aeruginosa*	Blood cultures were placed in an automated blood culture system. The automated microbiology growth and detection system detected microbial growth from blood specimens. TTP was routinely measured and automatically recorded by the machine	Retrospective cohort study	13	139	139 patients aged 18 years or older with the growth of P. aeruginosa in one or more blood cultures were included. When the bacteria grew in multiple blood cultures, the shortest TTP of blood cultures was recorded. Patients younger than 18 years, with polymicrobial bacteremia were excluded
2018, S. Simeon et al. [5]	France	*Staphylococcus aureus*	In each centre, approximately 10 mL of blood was inoculated into aerobic and anaerobic bottles by nurses using a standardized sterile technique. The BACTEC system (Becton Dickinson) was used in three sites, and BacT/Alert (bioMerieux) in one. TTP was defined as the time from the start of incubation to alert signal	Prospective cohort study	13.7	587	The VIRSTA prospective cohort study included 587 consecutive adult patients with SAB between April 2009 and October 2011 in eight tertiary-care university hospitals in France
2018, Shang-Yu Chen et al. [23]	Taiwan	*Nontyphoidal salmonella*	The standard practice of obtaining blood cultures in our hospital is to draw two sets of blood sample from separate limbs and inoculate into aerobic and anaerobic blood culture flasks (10 mL each, BD BACTECTM blood culture media). Blood samples were immediately sent to the microbiology laboratory and incubated in a BACTEC FX automated detection blood culture system. TTP refers to the time elapsed from the onset of incubation into the blood culture processor to the detection of bacterial growth, which was routinely reported to clinicians in our hospital. In patients with two or more sets of positive blood culture results, only the shortest TTP was used for analysis in this study	Retrospective study	10	206	206 patients > 20y/o with NTS bacteremia
2019, Niu, Xinrong et al. [26]	China	*Escherichia coli* and *Acinetobacter baumannii*	Isolation, identification, and sensitivity monitoring of microbiology were performed using the Vitek 2 automatic system (Meriere, France). Double-disc confirmation testing was used to detect ESBL + . E. coli ATCC 25,922 and A. baumannii ATCC 19,606 were used as controls	Retrospective study	7 days	89	A total of 118 patients, including 87 males and 31 females, with an average age of 52 ± 10.66, ranging from 25 to 78 years old, were collected
2019, Qinyuan Li [30]	China	*Streptococcus pneumoniae*	Approximately 3 ml of blood was inoculated into BACTEC plus aerobic bottles, which were then transported to the laboratory and incubated in an automated continuous monitoring system immediately. The Becton–Dickinson diagnostic systems were used for blood culture; it monitors CO2 production every 5 min by means of a fluorescent signal. Bottles with positive results were examined by Gram staining, and their contents were subcultured	Retrospective study	12	136	136 Children (< 18 y/o) with≧1 positive S. pneumoniae positive blood culture hospitalized in Children's Hospital of Chongqing Medical University from May 2011 to December 2017 were enrolled retrospectively
2019, Yuanyuan Li et el. [15]	China	*Staphylococcus aureus* bacteremia children	Approximately 3 mL of blood was inoculated into BACTEC plus aerobic bottles, which were then transported to the laboratory and immediately incubated in an automated continuous monitoring system. The BD diagnostic system was used for blood culture, which monitors CO2 production every 5 min by means of a fluorescent signal. Bottles with positive results were examined by Gram staining, and their contents were subcultured	Retrospective study	17	84	Children (< 18y/o) with≧ 1S. aureus positive blood culture hospitalized in Children's Hospital of Chongqing Medical University between 29 January 2014 and 29 August 2017 were enrolled retrospectively
2020, Huiting Xu et al. [21]	China	*Pseudomonas aeruginosa*	An approximately 3–5 ml of venous blood (> 0.5 mL for neonate) was inoculated into aerobic each BACTEC PLUS bottle and transported to the microbiological laboratory at any time of the day (24 h a day, 7 days a week). Blood cultures were processed employing the Becton–Dickinson diagnostic systems, which automated measured bacterial growth by continuously monitoring CO2 production in every 5 min, through a fluorescent sensor technology. Those positive cultures were subsequently subcultured after Gram staining	Retrospective study	18	52	52 children (< 18y/o) with P. aeruginosa bacteremia were enrolled
2020, Yufang Chen et al. [19]	China	*Escherichia coli*	At least two sets of blood samples, 20 ml each, were taken from separate venous sites and inoculated into aerobic and anaerobic culture bottles. These were loaded on a BACTEC 9120 automated detection blood culture system. All bottles giving a positive signal were examined by Gram staining, subcultured to blood agar medium and incubated for 18–48 h. The TTP for each bottle was defined as the time period from the start of incubation to the alert signal as documented by the monitoring system	Retrospective study	11	167	Adult inpatients (⩾18 years old) were considered eligible if they had a bloodstream infection with one or more blood culture positive for E.coli
2021, K. Michelson et al. [31]	Germany	*E. faecalis*	Blood cultures were drawn by medical professionals and sent to the Department of Microbiology, where they were incubated by Bactec FX automated incubation system. Bacterial growth was monitored via a fluorescent based detector measuring bacterial CO2 production. A positive blood culture was indicated by an automated alert signal. The shortest TTP of the first blood culture to be positive with Enterococcus spp. during one hospital stay was recorded. Every patient was only included once. Monomicrobial infections were defined as infection. that showed an exceeding majority of number of blood cultures with Enterococcus spp. For example, when three of four blood cultures were positive with Enterococcus spp. they were considered as monomicrobial. When two of four blood cultures were positive with Enterococcus spp. they were classified as polymicrobial and excluded from this analysis	Retrospective cohort study	4.35	54	477 patients were found to be positive for polymicrobial E-BSI. 244 patients with monomicrobial BSI and inappropriate antimicrobial therapy on the day of positive blood culture were analyzed
		Vancomycin sensitive *E. faecium*				135	
		Vancomycin resistant *E. faecium*				55	

*BSI* Bloodstream infection, *CO2* Carbon dioxide, *CoNS* Coagulase-negative staphylococcus, *CRBSI* Catheter-related bloodstream infections, *GNB* Gram’s negative bacteria, *GNB* Gram’s negative bacilli, *IE* Infective endocarditis, *NTS* Nontyphoidal Salmonella, *TTP* Time to positivity, *VCUMC* Virginia Commonwealth University Medical Center

**Fig. 2:**
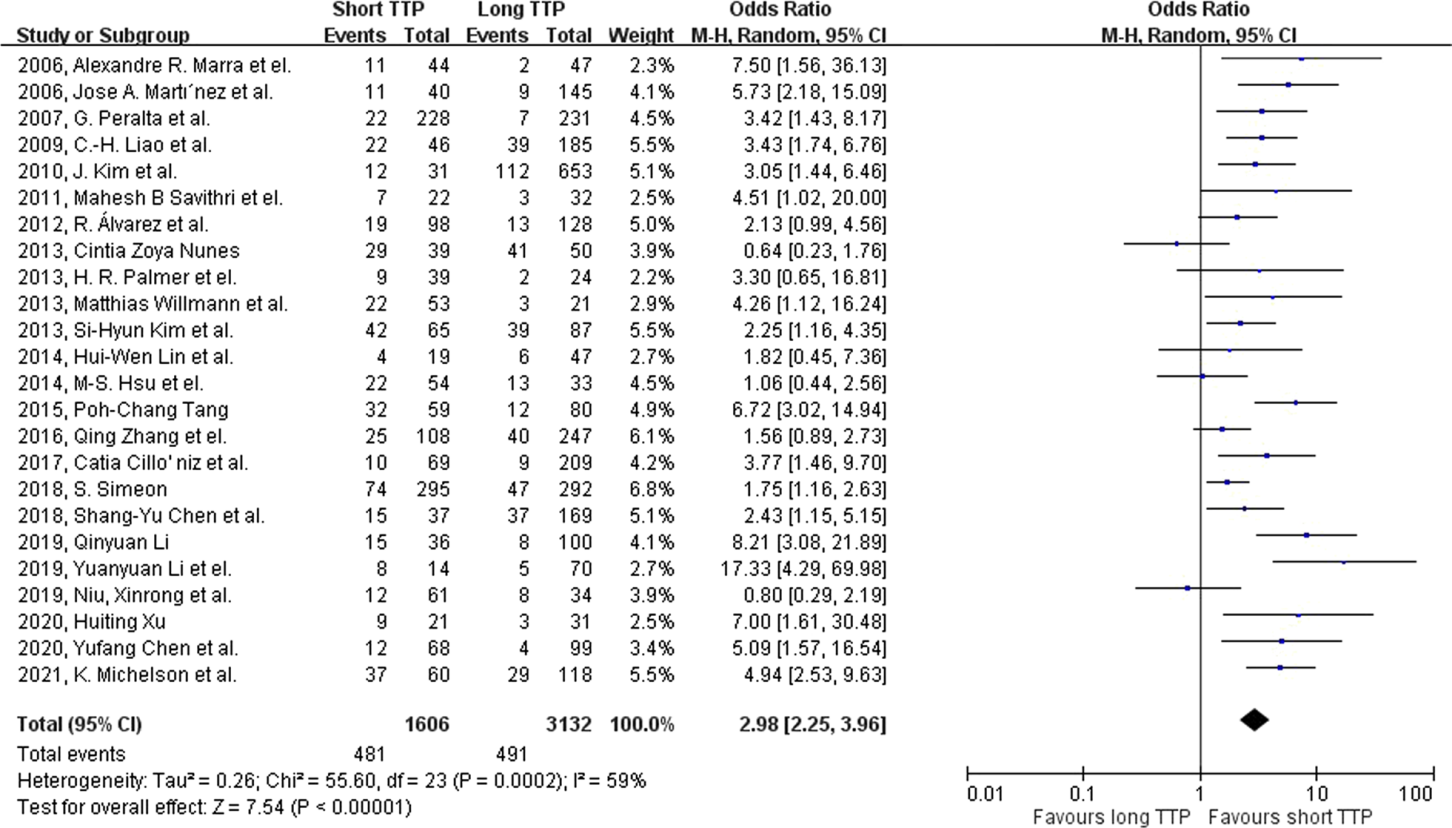
Forest plot showing the association between short TTP and patient mortality using the random-effects model. Events, population of mortality in both TTP groups; total, total population in both TTP groups

**Fig. 3:**
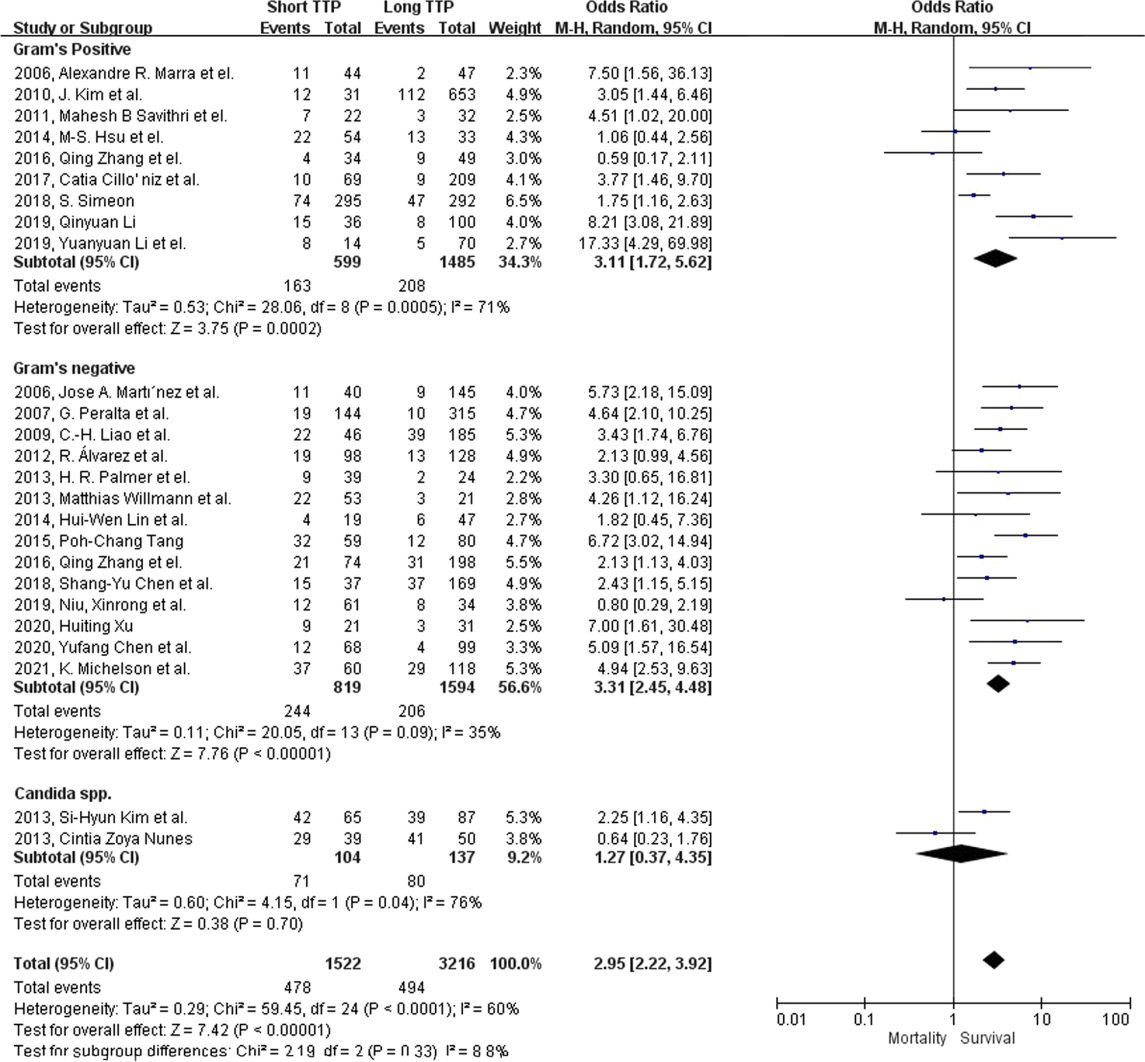
Forest plot showing the association between short TTP and patient mortality in Gram’s stain and Candida spp. subgroups. Events, population of mortality in both TTP groups; total, total population in both TTP groups

**Fig. 4:**
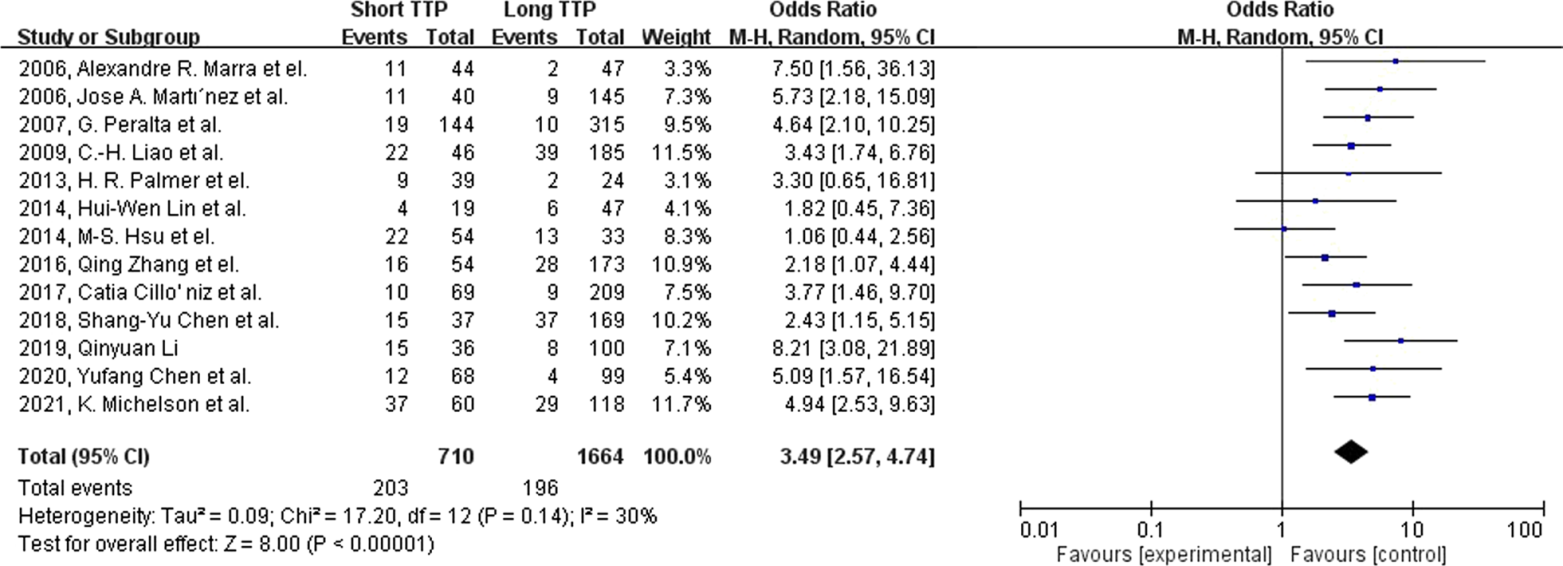
Forest plot showing the association between short TTP and patient mortality in studies with TTP cut-off value shorter than the median (12 h) using the random-effects model

Mortality is significantly associated with the short TTP group with an odds ratio of 2.98 (95% CI: 2.25–3.96, p-value < 0.001; Fig. 2). Moderate heterogenicity was found among our studies included in the mortality analysis group (${I}^{2}$=62%; Cochrane’s Q < 0.1). In sub-group analysis, short TTP is significantly correlated with mortality in *Gram's* positive and *Gram’s* negative bacterial group (Fig. 3), with an odds ratio of 3.11(95% CI: 1.72–5.62, p-value < 0.001), and 3.31(95% CI: 2.45–4.48, p-value < 0.001). However, in the *Candida* species group, mortality was not significantly correlated with short TTP (OR 1.27, 95% CI: 0.37–4.35, p-value = 0.7). In studies with a cut-off value shorter than the median, the odds ratio is 3.49 (95% CI: 2.57–4.74, p-value < 0.001; Fig. 4) with low heterogenicity (${I}^{2}$=30%; Cochrane’s Q = 0.14).

**Fig. 5:**
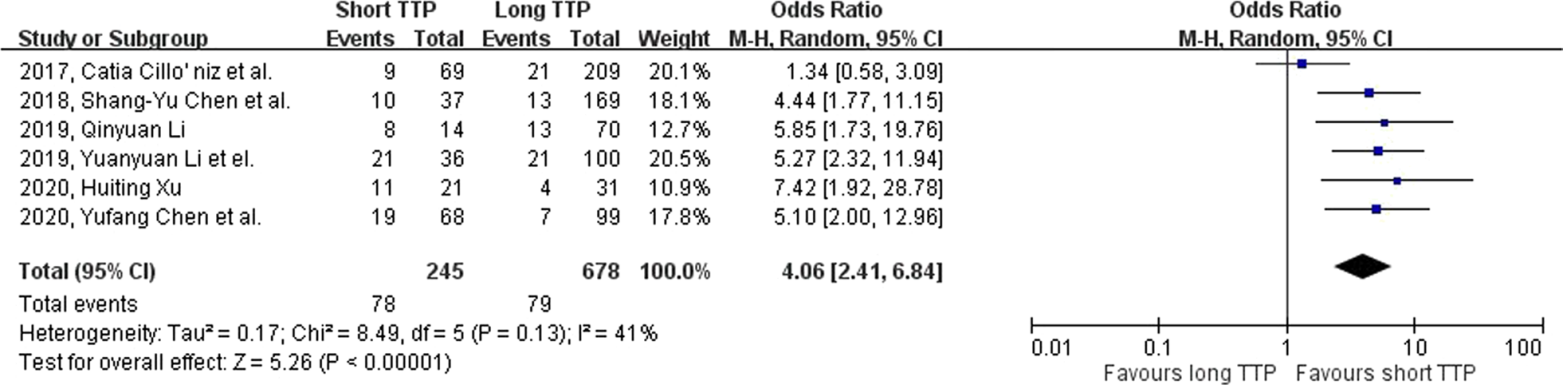
Forest plot showing the association between short TTP and septic shock in patients using the random-effects model. Events, population of septic shock in both TTP groups; total, total population in both TTP groups

**Fig. 6:**
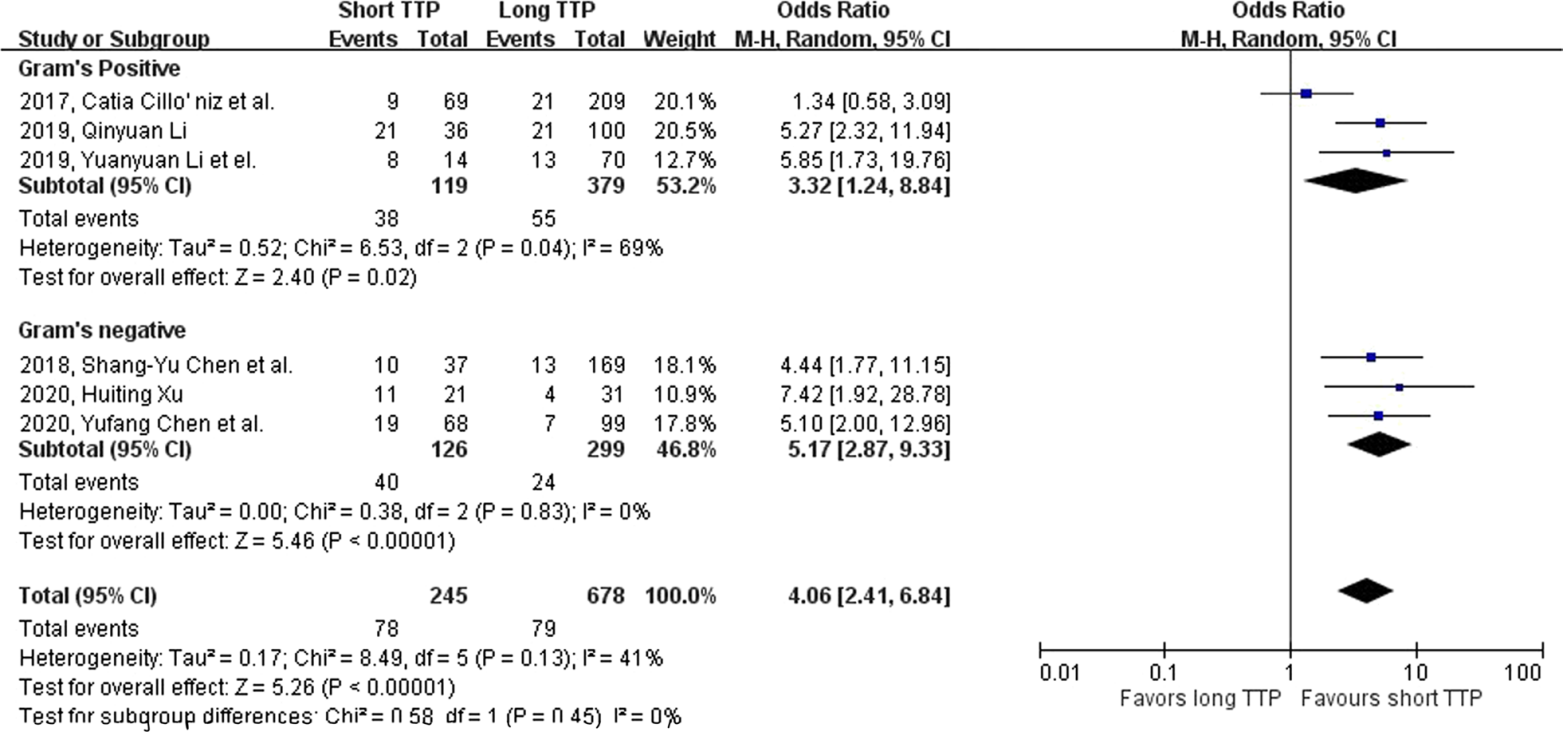
Forest plot showing the association between short TTP and septic shock in patients in Gram’s stain. subgroups. Events, population of mortality in both TTP groups; total, total population in both TTP groups

The odds ratio for developing septic shock in the short TTP group is 4.06 (95% CI: 2.41–6.84, p-value < 0.001; Fig. 5), and the heterogenicity is low (${I}^{2}$=41%; Cochrane’s Q = 0.13). Sub-group analysis (Fig. 6) showed a significant correlation of septic shock incidence rate with short TTP in both *Gram's* positive (OR 3.32, 95% CI: 1.24–8.84, p-value = 0.02) and *Gram's* negative (OR 5.17, 95% CI: 2.87–9.33, p-value < 0.001) bacterial group.

**Fig. 7:**
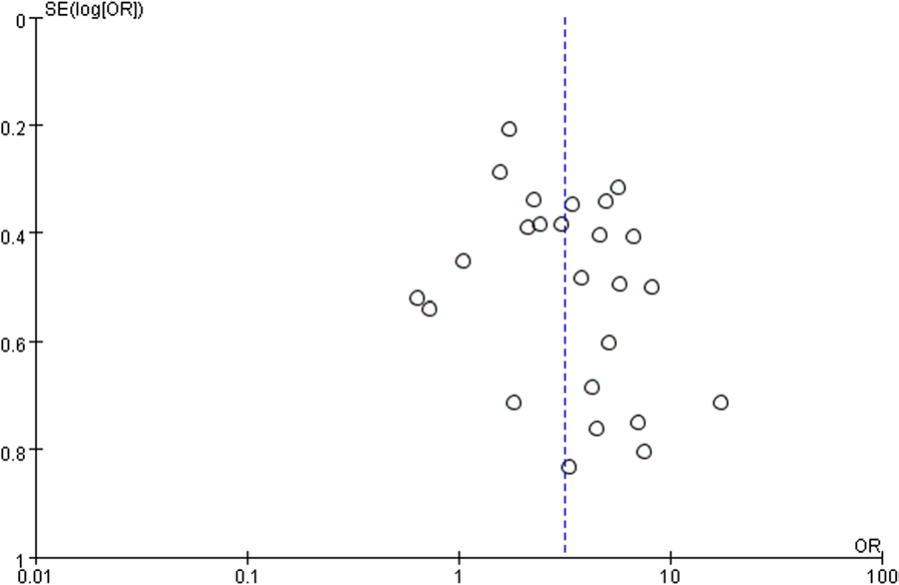
Funnel plot of studies included in the meta-analysis between short TTP and mortality

**Fig. 8:**
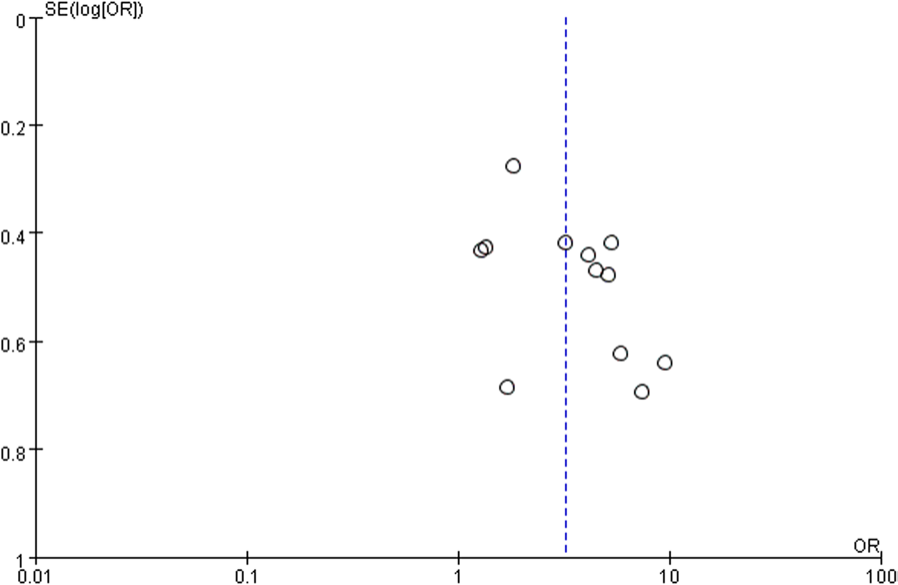
Funnel plot of studies included in the meta-analysis between short TTP and septic shock

Funnel plot analysis for the mortality group and the septic shock group showed grossly symmetrical with poor narrowing on large population studies (Figs. 7 and 8), which might represent publication bias and small sample size bias.

## Discussion

In this study, we collected numerous studies about the correlation between TTP and patient outcomes. Populations on mortality and septic shock in both TTP shorter and longer than the cut-off value defined in the article were extracted if available. Meta-analysis revealed a 2.98-fold higher mortality risk and a 4.06-fold higher risk for developing septic shock in the short TTP group. Sub-group analysis also showed short TTP to be an effective predictor of mortality and septic shock in different bacterial groups except for *Candida* species in mortality.

The utility of TTP has been investigated in several different aspects. Work has been done on the effectiveness of TTP for distinguishing bacterial species, differentiating the infection source, and predicting patient outcomes. TTP is proved to be an independent predictor of mortality and other categories of outcome in several studies [5–9]. Most of the previous studies reported a significant relationship between short TTP and mortality. This corresponds to the hypothesis that short TTP might be correlated to higher bacterial load [1, 9, 33], which results in higher mortality. Interestingly, mortality risk isn't always correlated with short TTP. In a retrospective study included in our analysis, mortality is associated with the long TTP group [8]. Six hundred eighty-four patients consisting of adult and pediatric *S. aureus* bacteremia revealed that TTP > 48 h was associated with higher 30-day mortality. The possible explanation might be because bacteria load in pediatric bacteremia is different in adult bacteremia, so merging two groups of *S. aureus* bacteremia might not be appropriate [34, 35]. Three recent articles reported a non-significant relationship between TTP and mortality, but the final result of the analysis was not affected. In a cohort enrolled 87 patients with *S. aureus* bacteremia, TTP of < 12 h was not significantly associated with mortality [13]. Only patients with bacteremia persisting for more than 48 h were included, and patients who died within 48 h were excluded, which might contribute to the phenomenon. The other two studies that showed TTP to be unpredictable for mortality might be because of the small population size included in their analyses. One study of 68 patients with nontyphoidal *Salmonella* bacteremia [22], and another is a prospective observational study about Gram-negative bacilli bacteremia with 63 patients enrolled in the final analysis [28].

Most bacterial groups we analyzed revealed a significant relationship among the subgroup analysis of short TTP and mortality. However, our analysis reported a non-significant result (p-value = 0.7) in the *Candida* species group. We included two studies in *Candida* species subgroup analysis. One is a retrospective study including 152 patients [25]. In this article, short TTP is independently associated with an increased 6-week mortality rate in patients with candidaemia. Another is a separate cohort study including 89 adult patients with *C. Albican*s bacteremia infection [24]. Interestingly, this study showed that the longer the TTP, the higher the mortality risk. The result could be the etiology of candidaemia, general patient health included in this study, or the volume inoculated into the blood culture bottles. To our knowledge, these two articles are the only articles discussing TTP and patient outcome in candidaemia and having available data for us to extract and analyze. Other articles about candidaemia and TTP mainly discuss the relationship between TTP and different *Candida* species or different culture sites.

The predictive capability of short TTP for septic shock events is also evaluated in our study. Our analysis revealed that short TTP could indicate septic shock, which is in line with previous studies. The result of our subgroup analysis is also consistent with previous reports.

We conducted another statistical analysis, including studies with cut-off values shorter than the median of cut-off values in our study to eradicate the effect of the varied cut-off values and the heterogenicity noticed among the articles in the mortality group. The correlation remains significant with short TTP with a higher odds ratio (3.49, 95% CI: 2.57–4.74, p-value < 0.001) compared with the original analysis (2.98, 95% CI: 2.25–3.96, p-value < 0.001) but with lower heterogenicity in the latter analysis. This result emphasizes the correlation between short TTP and mortality.

There are some limitations to our analysis. First, there might be some notable bias in our study. Although the funnel plot in our analysis is symmetrical, the narrowing is insufficient among the large population studies, which might represent possible publication bias or reporting bias. Second, heterogenicity is noted in mortality analyses. Cochrane's Q value < 0.1 and an ${I}^{2}$ value of 62% were noted is mortality rate analysis. This could be attributable to the variation of cut-off values in each study. Each article we included reported an individual cut-off value for TTP. Meaning every short/long TTP group is defined under different cut-off value, which could cause underlying bias and poor utility of the result of our analysis. Thus, a cut-off value would be crucial for TTP to be clinically applicable, although the prediction with short TTP on patient mortality and septic shock is confirmed. However, we offset a part of the bias by performing another analysis including studies with cut-off values shorter than the median. The heterogenicity is lesser and the odds ratio is larger in this analysis. Third, merging pediatric and adult patients in our study might also contribute since children and adults have different bacterial loads and different blood culture volumes inoculated into the blood culture bottle [36, 37]. Fourth, there are notable confounders in our studies. Most of them are not included in our analysis due to the small number of articles discussing the association of these factors with TTP. All of our studies did not exclude the effect of confounding factors such as the delay of bottle loading, the difference between each blood culture system, administered antimicrobials, site of infection, time to start of antimicrobials, and the volume of blood cultured [10], which might lead to hampered external validity and difficulties for TTP application in respective centers. Thus, further analyses with studies considering more confounder effects and more articles included would be necessary. Fifth, the meta-regression analysis we performed revealed no significant result; this might be because of the small number of articles performing multivariate analysis with TTP and patient outcome. Last but not least, a systemic review on if a short time to positivity is associated with high markers of inflammation is necessary for our article to prove that hyper-inflammation is the explanation of unfavorable outcomes. Only few articles discuss the correlation between inflammation marker and TTP.

## Conclusion

In conclusion, this meta-analysis confirmed that a short TTP might be predictive for patient mortality and septic shock. Possible infection etiology and bacteria genre should take into concern while trying to apply this result clinically.

## Supplementary Information

**Additional file 1.** Search terms and connectors (AND/OR) for literature search.

**Additional file 2.** CASP quality assessment.

## Data Availability

The datasets used and/or analysed during the current study are available from the corresponding author on reasonable request.

## Abbreviations

BSI: Bloodstream infection
CI: Confidence interval
CASP: Critical appraisal skills programme
CO2: Carbon dioxide
CoNS: Coagulase-negative staphylococcus
CRBSI: Catheter-related bloodstream infections
GNB: Gram’s negative bacteria
GNB: Gram’s negative bacilli
IE: Infective endocarditis
MeSH: Medical subject headings
NTS: Nontyphoidal Salmonella
OR: Odds ratio
PRISMA: Preferred reporting items for systematic reviews and meta-analyses
TTP: Time to positivity
VCUMC: Virginia Commonwealth University Medical Center
