# Supplementary material for: Short time to positivity of blood culture predicts mortality and septic shock in bacteremic patients: a systematic review and meta-analysis
Source: BMC Infect Dis. 2022 Feb 10;22:142. doi: 10.1186/s12879-022-07098-8 (PMC8830084; doi:10.1186/s12879-022-07098-8)
Supplement: Supplementary file 1 — Additional file 1. Search terms and connectors (AND/OR) for literature search. [file 12879_2022_7098_MOESM1_ESM.docx]

|  | Did the study address a clearly focused issue? | Was the cohort recruited in an acceptable way? | Was the exposure accurately measured to minimise bias? | Was the outcome accurately measured to minimise bias? | Have the authors identified all important confounding factors? | Have they taken account of the confounding factors in the design and/or analysis? | Was the follow up of subjects complete enough? | Was the follow up of subjects long enough? | Do you believe the results? | Can the results be applied to the local population? | Do the results of this study fit with other available evidence? | Score / 12 questions |
| --- | --- | --- | --- | --- | --- | --- | --- | --- | --- | --- | --- | --- |
| 2006, A. R. Marra et el. [13] | Y | Y | Y | Y | N | Y | Y | Y | Y | Y | Y | 11 |
| 2006, J. A. Martı´nez et al. [16] | Y | Y | N | Y | N | Y | Y | Y | Y | Y | Y | 10 |
| 2007, G. Peralta et al. [17] | Y | Y | Y | Y | N | Y | Y | Y | Y | Y | Y | 11 |
| 2009, C.-H. Liao et al. [32] | Y | Y | Y | Y | Y | Y | Y | N | Y | Y | Y | 11 |
| 2010, J. Kim et al. [8] | Y | Y | Y | N | Y | Y | Y | Y | Y | Y | Y | 11 |
| 2011, M. B. Savithri et el. [29] | Y | Y | Y | Y | CT | Y | Y | N | Y | Y | Y | 9 |
| 2012, R. Álvarez et al. [18] | Y | Y | Y | Y | N | Y | Y | Y | Y | Y | Y | 11 |
| 2013, Cintia Zoya Nunes [24] | Y | Y | Y | Y | N | N | Y | CT | Y | Y | N | 8 |
| 2013, H. R. Palmer et el. [28] | Y | Y | Y | Y | N | Y | Y | Y | CT | Y | N | 9 |
| 2013, M. Willmann et al. [9] | Y | Y | Y | Y | N | Y | Y | Y | Y | Y | Y | 11 |
| 2013, Si-Hyun Kim et al. [25] | Y | Y | Y | Y | N | Y | Y | Y | Y | Y | N | 10 |
| 2014, Hui-Wen Lin et al. [22] | Y | Y | Y | Y | Y | Y | Y | CT | CT | Y | N | 9 |
| 2014, M-S. Hsu et el. [14] | Y | Y | Y | Y | Y | Y | Y | CT | Y | Y | N | 10 |
| 2016, Qing Zhang et el. [27] | Y | Y | Y | Y | N | Y | CT | CT | Y | Y | Y | 9 |
| 2017, Catia Cillo' niz et al. [7] | Y | Y | Y | Y | N | Y | Y | Y | Y | Y | Y | 11 |
| 2017, Poh-Chang Tang et al. [20] | Y | Y | Y | Y | Y | Y | Y | CT | Y | Y | Y | 11 |
| 2018, S. Simeon et al. [5] | Y | Y | N | Y | N | Y | Y | Y | Y | Y | Y | 10 |
| 2018, Shang-Yu Chen et al. [23] | Y | Y | Y | N | Y | Y | Y | N | Y | Y | Y | 10 |
| 2019, Niu, Xinrong et al. [26] | Y | CT | Y | Y | N | N | Y | Y | CT | CT | CT | 6 |
| 2019, Qinyuan Li et al. [30] | Y | Y | Y | Y | N | N | Y | CT | Y | Y | Y | 8 |
| 2019, Yuanyuan Li et el. [15] | Y | Y | Y | Y | N | Y | Y | CT | Y | Y | Y | 9 |
| 2020, Huiting Xu [21] | Y | Y | Y | Y | N | Y | Y | CT | Y | Y | Y | 9 |
| 2020, Yufang Chen et al. [19] | Y | Y | Y | Y | N | Y | Y | CT | Y | Y | Y | 9 |
| 2021, K. Michelson et al. [31] | Y | Y | Y | Y | Y | Y | Y | Y | Y | Y | Y | 12 |

Y=Yes, N=No, CT=Can’t Tell
