# Supplementary material for: Short time to positivity of blood culture predicts mortality and septic shock in bacteremic patients: a systematic review and meta-analysis
Source: BMC Infect Dis. 2022 Feb 10;22:142. doi: 10.1186/s12879-022-07098-8 (PMC8830084; doi:10.1186/s12879-022-07098-8)
Supplement: Supplementary file 2 — Additional file 2. CASP quality assessment. [file 12879_2022_7098_MOESM2_ESM.pdf]

We used the keyword of "time to positivity", "time to blood culture positivity", “time to culture positivity” combined with “Mortality”, “outcome”, “outcome assessment”, “patient outcome”, “septic shock”, “severe sepsis” to search the database of Pubmed, EMBASE, CINAHL, Web of Science, Cochrane library. The specific keyword use in each database are listed below.

Pubmed	(((("time to positivity") OR ("time to culture positivity")) OR ("time to blood culture positivity")) AND (("Shock, Septic"[Mesh]) OR ("Mortality"[Mesh]) OR ("Sepsis"[Mesh])) AND (("Prognosis"[Mesh]) OR ("Outcome Assessment, Health Care"[Mesh]))
EMBASE	'time to positivity' OR 'time to culture positivity' OR 'time to blood culture positivity' AND 'outcome'/exp OR 'outcome assessment'/exp OR 'prognosis'/exp OR 'patient outcomes'/exp
CINAHL	(((MH "Outcome Assessment") OR (MH "Fatal Outcome") OR (MH "Treatment Outcomes")) OR ((MH "Prognosis") OR (MH "Clinical Assessment Tools")) OR ((MH "Mortality") OR (MH "Maternal Mortality") OR (MH "Hospital Mortality") OR (MH "Child Mortality") OR (MH "Infant Mortality")) OR ((MH "Neonatal Sepsis") OR (MH "Sepsis") OR (MH "Systemic Inflammatory Response Syndrome") OR (MH "Fungemia") OR (MH "Bacteremia"))) AND (TX "time to positivity" OR TX "time to blood culture positivity" OR TX "time to culture positivity")
Web of Science	"time to positivity" OR "time to blood culture positivity" OR "time to culture positivity" AND outcome OR outcome assessment OR prognosis OR patient outcome AND mortality OR mortality rate OR mortality risk OR sepsis OR severe sepsis OR septic shock
Cochrane Library	("time to positivity") OR ("time to blood culture positivity") OR ("time to culture positivity") AND ((outcome) OR (outcome assessment) OR (patient outcome) OR (prognosis) OR (mortality) OR (sepsis) OR (severe sepsis) OR (septic shock))
